# STRESS-testing clinical activity and outcomes for a combined prison in-reach and court liaison service: a 3-year observational study of 6177 consecutive male remands

**DOI:** 10.1186/s13033-016-0097-z

**Published:** 2016-10-11

**Authors:** Conor O’Neill, Damian Smith, Martin Caddow, Fergal Duffy, Philip Hickey, Mary Fitzpatrick, Fintan Caddow, Tom Cronin, Mark Joynt, Zetti Azvee, Bronagh Gallagher, Claire Kehoe, Catherine Maddock, Benjamin O’Keeffe, Louise Brennan, Mary Davoren, Elizabeth Owens, Ronan Mullaney, Laurence Keevans, Ronan Maher, Harry G. Kennedy

**Affiliations:** 1 National Forensic Mental Health Service, Central Mental Hospital, Dundrum, Dublin, 14 Ireland; 2 Department of Psychiatry, Trinity College, Dublin, Ireland; 3 Irish Prison Service, Dublin, Ireland

**Keywords:** Prison psychiatry, Court diversion, Court liaison, Screening, Clinical pathways, Risk assessment

## Abstract

**Background:**

People with major mental illness are over-represented in prison populations however there are few longitudinal studies of prison in-reach services leading to appropriate healthcare over extended periods.

**Aims:**

We aimed to examine measures of the clinical efficiency and effectiveness of a prison in-reach, court diversion and liaison service over a 3 year period. Secondly, we aimed to compare rates of identification of psychosis and diversion with rates previously reported for the same setting in the 6 years previously. We adopted a stress testing model for service evaluation.

**Method:**

All new male remand committals to Ireland’s main remand prison from 2012 to 2014 were screened in two stages. Demographic and clinical variables were recorded along with times to assessment and diversion. The DUNDRUM Toolkit was used to assess level of clinical urgency and level of security required. Binary logistic regression was used to assess factors relevant to diversion.

**Results:**

All 6177 consecutive remands were screened of whom 1109 remand episodes (917 individuals) received a psychiatric assessment. 4.1 % (95 % CI 3.6–4.6) had active psychotic symptoms. Levels of self-harm were low. Median time to full assessment was 2 days and median time to admission was 15.0 days for local hospitals and 19.5 days for forensic admissions. Diversion to healthcare settings outside prison was achieved for 5.6 % (349/6177, 95 % CI 5.1–6.3) of all remand episodes and admissions for 2.3 % (95 % CI 1.9–2.7). Both were increased on the previous period reported. Mean DUNDRUM-1 and DUNDRUM-2 Triage Security Scores were appropriate to risk and need.

**Conclusions:**

We found that a two-stage screening and referral process followed by comprehensive assessment optimised identification of acute psychosis. The mapping approach described shows that it is possible for a relatively small team to sustainably achieve effective identification of major mental illness and diversion to healthcare in a risk-appropriate manner. The stress-testing structure adopted aids service evaluation and may help advise development of outcome standards for similar services.

## Background

Prisons have been described as representing a ‘rare public health opportunity’ for identifying and managing major mental illness in young men [1] and can provide a focal point for arranging diversion to healthcare [2]. In a review of research on the mental health of prisoners, including a number of meta-analyses, Fazel et al. [3] identified the need for longitudinal studies of mental health in prisoners. There remains a need to determine and define the variables measuring the effectiveness of prison mental health services. Further, there is limited research describing sustainable clinical pathways over extended periods for persons receiving mental health care in prisons.

Cross-sectional prevalence rates of psychotic illness in prison populations have been estimated at ten times the community rate [4, 5]. Fazel and Seewald found a pooled prevalence of psychotic illness (including psychosis, schizophreniform disorders and manic episodes) of 3.6 % in a systematic review and meta-analysis of studies involving 33,588 prisoners from 24 countries [6]. Still higher rates have been found in studies of male remands in Ireland [7] and England and Wales [8]. Curtin et al. [9] found 3.8 % (95 % CI 2.2–6.6 %) of a series of 313 male remands in Ireland had a current diagnosis of psychotic disorder (including schizophrenia, psychotic mood disorders, substance-induced psychosis and other organic psychoses).

Pakes and Winstone audited areas of practice in 101 prison mental health services across England [10]. They noted that while screening and mental health assessment was mainly performed adequately, proactive screening was sporadic and services were often unstructured. In particular they reported a lack of coherence in the way data was collected in different sites. Pillai et al. [11], showed that the introduction of a formalised model of care was followed by an increase in the proportion of referrals taken onto clinical caseloads in remand and sentenced settings over a six-year period (2010–2015).

The Bradley Report [12] echoed the Reed Report of 1992 [13] in recommending improved coordination and integration of prison mental health care and diversion services. Quality standards have now been developed as part of a quality network for prison mental health services to support improve and standardise prison mental health services in a measureable way [14]. These standards provide for qualitative comparisons but do not in their current iteration provide for quantitative measurement and comparison of specific outcomes.

Coid and Ullrich [5] have stated that psychiatric services *“fail to identify psychotic prisoners and provide after care (pg 99)”* and suggested that *“proposals to divert more offenders with severe mental illness to mental health services … may currently be unfeasible (pg 107)”* due to difficulties in identification, limited resources and the need for the public to be reassured that diversion is risk-appropriate, with diversion of high-risk patients to more secure settings.

Stress testing refers to a deliberate process to determine the stability of a given system or entity. The approach is used in a range of settings including financial systems, information technology and engineering to confirm that intended specifications are being met and to help determine modes of failure [15, 16]. An effectively functioning prison mental health in-reach service should be able to ‘count in and count out’ those using the service, identifying those with the most severe acute symptoms and arrange transfer of care at the point of exit. The service should also allocate patients to the appropriate level of care according to their assessed need (Flynn et al. [23]). This is particularly important for remand settings where a rapid turnover of prisoners can risk individuals with major mental illness being lost to follow up. Inability to achieve or effectively measure such outcomes may reflect a service under stress, and may help advise resource requirements or system recalibration. In prison settings, the greatest turnover is in remand settings, where higher rates of mental illness have been identified [6–9]. For patients subsequently transferred to sentenced prisons, much of the work of comprehensive assessment including taking histories, collateral gathering and care-planning will be completed in remand settings.

Our research group has previously described a combined prison in-reach, court diversion and liaison scheme in Ireland’s main remand prison which identified the presence of active psychotic symptoms following committal among 20,084 consecutive male remands at rates in keeping with epidemiologically predicted rates, and diverted 572 cases from prison to healthcare over a 6 year period from 2006 to 2011 [17]. Limitations of that study included failure to present a number of key outcome measures, including time to achieve assessment and risk-appropriateness of diversions to different levels of security. We undertook to address these limitations in further research with an approach structured to enable intra-service comparison over time, comparison with similar services and with existing epidemiological evidence.

## Aims

The aim of this study was to describe the longitudinal activity of a psychiatric in-reach, court liaison and diversion service in a male-only remand prison over a 3 year period, using quantitative routine outcome measures of service outcomes. It was intended that the routine outcome measures used would enable comparisons of service activity over time for our service and act as a basis for comparison with activity and outcomes of similar services elsewhere.

The format we used is divided into six domains: screening, identification and description of service caseload, transfer of care, risk-appropriateness of diversions, efficiency, self-harming behaviours and service mapping. These domains are summarised by the acronym STRESS-Testing. The domains and associated aims of the study are laid out in Table 1, below.

**Table 1:** STRESS-testing: screening, identification and description of service caseload, transfer of care, risk-appropriateness of diversions, efficiency and productivity, self-harming behaviours and service mapping

	Domain	Aim
1.	Screening, Identification and caseload description	How many remands were screened? How many were assessed and taken onto the team caseload? Is the caseload over time described in terms of diagnosis, co-morbid conditions and offence type? Is the caseload described in terms of other factors including homelessness, whether or not known to have a past history of self harm and whether or not known to have previous contact with psychiatric services outside prison Is the service identifying persons with the most severe acute symptoms, such as active psychotic symptoms at rates in keeping with expected rates based on the existing epidemiological literature?
2.	Transfer of care	How many were diverted from the criminal justice system to mental health treatment settings?
3.	Risk-appropriateness of diversions	Were diversions to forensic inpatient settings, to general psychiatric inpatient settings and to outpatient settings justifiable in terms of assessed security requirements and clinical urgency?
4.	Efficiency and productivity	What was the delay from committal screening to first comprehensive assessment? Were persons identified as actively psychotic seen more rapidly than persons without acute psychotic symptoms? What was the delay from committal and first assessment to diversion? How many cases were managed and diversions achieved per whole time equivalent employed?
5.	Self-harm	How many persons deliberately harmed themselves in custody over the study period?
6.	Service mapping	Can the service ‘map’ the flow of all patients through the system, with outcomes at the point of discharge and times to those outcomes? Can the service map subsequent outcomes for persons admitted to the ‘parent’ forensic psychiatric unit?
7.	Testing	How did the above activity and outcome data compare with previously published findings for the same service in the six years preceding this three-year study? How did outcomes compare year on year within the same service?

## Methods

### Location and context

Cloverhill, a male-only prison, is Ireland’s main remand facility and receives a majority of remands from Ireland’s courts including those in and around Dublin, Ireland’s most populous conurbation. During the period 2006–2011, the prison received approximately 60 % of all remands nationally [17]. The remaining 40 % of remands nationally were committed to three other mixed remand and sentenced facilities in the south and west of the country. From 2nd December 2013 persons who received sentences for non-indictable offences from a single busy district court based adjacent to the prison, were also committed to Cloverhill Prison.

A multidisciplinary mental health team has been provided 5 days a week (Monday–Friday) to the prison since 2006 to enable systematic identification and diversion to healthcare of persons with mental illness. The team is called the Prison In-reach and Court Liaison Service (PICLS).

Fully staffed, the team consisted of a Consultant Psychiatrist, two to three psychiatric trainees and three forensic mental health nurses. Not all posts were filled throughout the 3 year study period. The team received secretarial support from the Central Mental Hospital. PICLS staff members are employed by the National Forensic Mental Health Service, a part of the Health Service Executive, Ireland’s public mental health service funded by the Department of Health. In January 2014 a housing support worker funded by the Genio Trust joined the service to assist in arranging accommodation on release for persons seen by the mental health team.

The prison was also attended by general nursing staff and general practitioners, employed by the Irish Prison Service, an agency of the Department of Justice. A number of other in-reach services also attended the prison including addiction psychiatry, addiction counselling, infectious disease services, probation and welfare and chaplaincy services.

The legal structure for mental healthcare in Ireland has been summarised elsewhere [17]. Gardai (Irish Police) may make applications for admission to psychiatric facilities under Section 12 of the Mental Health Act 2001. Persons may be transferred from prison to the Central Mental Hospital (CMH), Ireland’s only designated forensic psychiatric inpatient facility, under Section 15 of the Criminal Law (Insanity) Act 2006. Persons may also be admitted under this Act following findings of unfitness to stand trial (Section 4) or not guilty by reason of insanity (Section 5). There is currently no other specific legislation enabling court diversion to general psychiatric inpatient or community settings, but diversion can take place within bail and probation legislation and existing mental health law.

### Ethical approval

The research protocol for this study was approved by the National Forensic Mental Health Service Research, Audit, Ethics and Effectiveness Committee. Only anonymised information from a large sample was analysed and presented in the current study. Data utilised was routinely collected for the service’s evaluation and annual reports which have become more comprehensive as the service has developed. No individual patient data has been presented.

### Data analysis

Anonymised information was analysed using SPSS 20 [18]. Confidence intervals for proportions were calculated using the Epi-Tools program [19].

### Service process

All persons remanded by court to the prison were screened on committal, generally within 2 h of their reception by prison general nursing staff using a 7-item variant of the screening tool developed by Grubin et al. [20]. Automatic referrals were thus generated. All committals were also seen within 24 h by a prison General Practitioner. Where there were concerns regarding acute mental or physical health issues, persons were transferred to a vulnerable person’s unit called D2 wing, under conditions of special observation. Other new committals were initially placed in a “first night” area of the prison prior to transfer to normal wings within the prison.

On the first working morning following committal, PICLS team members reviewed the nurse screening results and the result of the General Practitioner’s assessment where this had been completed. In this ‘second stage screening’, team members also scrutinised medical and psychiatric electronic case notes in relation to previous committal episodes. In addition, the team accepted referrals from the courts, prison medical and nursing staff, chaplaincy services and regularly received requests for review from other sources, including family members, probation services and legal representatives.

Persons identified as requiring psychiatric assessment had a detailed history taken by pairs of interviewers from the PICLS team. Assessments were supplemented by collateral information. This included review of charges. Where a mental health problem was identified, collateral was sought from agencies including the person’s community General Practitioner, community psychiatric services, police and family members where available.

Communication with relevant forensic and community mental health services began at the point of initial assessment, including a written letter and collateral-gathering. For those requiring admission to hospital, decision-making regarding clinical urgency and the level of security required was assisted by the DUNDRUM Toolkit, a validated structured professional judgement instrument [21]. This was used to assist in deciding whether to refer to forensic or general hospital settings or outpatient care. For such individuals, structured reports were prepared for the court hearing, either voluntarily or on request from the court. These reports included a detailed history, with conclusions regarding diagnosis, fitness to be tried, a preliminary opinion regarding responsibility and advice regarding treatment arrangements in the event of custodial and non-custodial disposal. PICLS staff members appeared in court to provide oral evidence, to liaise with defence and prosecution legal staff and assist in bringing persons to hospital where required.

Care plans including contingency planning for custodial and non-custodial disposals were updated regularly for each patient at fortnightly multi-disciplinary meetings and in advance of the next court date. This meeting was also used to confirm and update diagnosis and record clinical outcomes for all patients who had been discharged, diverted to healthcare elsewhere or transferred to other prisons. This “rolling record” process enabled annualised and aggregated reports of the service’s activity.

Weekly interagency meetings were attended by prison medical and nursing staff, chaplaincy, probation services, the prison governor and addiction counselling services to discuss persons in the high support unit, identify vulnerable patients elsewhere in the prison including those at risk of self-harm and those who had self-harmed in the prison in the previous week.

### Study method

#### Screening, identification and caseload description

All new remands from court to Cloverhill Prison during the 3 years from 1st January 2012 to 31st December 2014 were screened as described above. Remand episodes were defined as committals from the courts on remand, trial, deportation and extradition. Sentenced episodes (unless also remanded) were excluded from the analysis to enable comparisons with other remand settings and previously published data from the same setting.

Data in tables has been presented where possible in binary (yes/no) format to facilitate comparisons over time and with other similar services. As far as possible, data has been presented as it relates to individuals as well as to individual remand episodes.

Demographic and clinical variables recorded by the PICLS team included nationality, homelessness, lifetime history of psychosis, history of substance misuse, history of deliberate self-harm and history of contact with psychiatric services outside prison. These were based on screening, assessment and collateral. Offence type was recorded as the most serious current charge recorded on committal. A violent offence was defined as an act of physical violence on a person and included homicide, assault, robbery, aggravated burglary, contact sexual offences, false imprisonment, driving offences involving injury to others and arson where there was a possibility of injury to others. Homelessness was defined as not having regular accommodation, rough sleeping or residence in homeless shelters identified at the time of committal.

ICD-10 [22] diagnoses were recorded in case notes following assessment by PICLS team members based on clinical interviews and review of past medical and psychiatric case records from prison and community sources. We recorded the presence or absence of identified active psychotic symptoms following committal, defined as current hallucinations, delusions and/or thought disorder and assessed repeatedly over time.

#### Transfer of care

Final disposal outcome was recorded for all committal episodes. This was defined as arrangements put in place for transfer of care at the point of discharge from the PICLS team. Diversion was defined as transfer from the criminal justice system to mental health care. Possible diversion outcomes were forensic admission, general psychiatric admission, and community outpatient treatment arranged. Community outpatient treatment included general psychiatric outpatient care, addiction psychiatry outpatient or rehabilitation care, psychiatric outpatient services for homeless persons and primary care follow-up. Non-diversion options were transfer to in-reach mental health care in another prison, discharge to prison GP and/or addiction services, deportation/extradition to another jurisdiction or remaining on the PICLS case-load. For those persons remanded during the 3 year study period ending 31st December 2014, final transfer of care outcomes were the transfer of care arranged by 9th April 2015.

#### Risk-appropriateness of diversions

Binary measures of whether the person was charged with a violent offence and the presence of active psychotic symptoms on committal, enable a crude estimate of risk-need appropriateness (whether transfer to a given level of therapeutic security is necessary) and are presented here.

The DUNDRUM Toolkit [23] is a suite of four structured professional judgement instruments intended to provide a validated and transparent means of making decisions about admission, transfer and discharge in forensic mental health services. DUNDRUM-1 [23, 24] rates security needs. DUNDRUM-2 [25] rates urgency of need for admission and helps prioritise persons on waiting lists. The eleven security and six urgency items are each rated on a five-point scale (0–4). For each item, ‘4’ indicates a need for high therapeutic security, ‘3’ for medium security, ‘2’ for low security and ‘1’ for open settings. The sum score is divided by the number of items to yield a mean score which is always between 0 and 4. A mean DUNDRUM-1 score greater than 3 would guide a need for high therapeutic security, between 2 and 3 would guide towards medium therapeutic security, between 1 and 2 would guide towards acute low therapeutic security (often referred to as psychiatric intensive care) while lower scores indicate open hospital ward or community settings. These item and scale scores guide but do not bind the clinical decision maker in individual cases. The mean scores for groups are useful guides to the appropriateness of patient placement from a risk-need-appropriateness perspective to ensure proportionality and safety.

DUNDRUM-1 and DUNDRUM-2 mean scores were calculated on a weekly basis for persons placed on waiting lists. The mean scores presented for persons diverted to forensic, general inpatient and general outpatient settings was the score as measured in the week prior to the outcome.

#### Efficiency

Timeframes from committal to first clinical assessment and diversion outcomes were calculated in days, such that 0 days means assessed/diverted on the same day and 1 day means assessed or diverted the next day. Therefore if a person was remanded on a Friday and first assessed on a Monday, the time to assessment was counted as 3 days. Medians were calculated to moderate the distorting effect of outliers, although means were also calculated. Remand episodes taken onto the clinical caseload per team member were also calculated.

#### Self-harm

Episodes of self-harm were recorded in prison healthcare medical records by prison staff. These episodes were cross-checked each week at a formal interagency meeting with bimonthly review at interagency suicide prevention meetings.

#### Service mapping

All remands were mapped in flow-chart format to show the pathway through care from the point of remand to final transfer of care and the time to assessment and diversion to mental health services outside prison. We also recorded subsequent placement arrangements for persons admitted to the Central Mental Hospital to 9th April 2015.

#### Testing

We present previously published activity and outcome data for remands to Cloverhill during the 6 year timeframe 2006–2011 with confidence intervals, to enable comparisons of measures of clinical activity and service outcomes in the subsequent years 2012–2014.

For those on the 2012–2014 caseload diverted to healthcare settings outside prison, we calculated proportions for each outcome who were actively psychotic, previously known to psychiatric services outside prison, Irish, homeless, those who had a final ICD-10 diagnosis of F20–F31 (schizophreniform and bipolar disorders), those with a history of substance misuse, those with history of deliberate self harm and those charged with a violent offence.

Binary logistic regression with any mental health diversion as the outcome was used to test strengths of association (odds ratios) and also to test which amongst these variables accounted for most of the association. All variables associated with psychiatric admission were also entered into a binary logistic regression model.

## Results

### Screening, identification and caseload description

#### Service activity

There were 6177 remand committals (all males) to Cloverhill Prison during the period 1st January 2012 to 31st December 2014, all of whom were screened (Table 2). This constituted 60.9 % (6177/10,148; 95 % CI 59.9–61.8 %) of male remand episodes nationally and 12.6 % of all prison committals (male and female, remand and sentenced) in the state during the years 2012–2014 (6177/48,916; 95 % CI 12.3–12.9 %) [25–27].

**Table 2:** All committals nationally, male remand committals nationally, male remands to Cloverhill, Number screened and taken onto PICLS caseload for years 2012–2014

	Total 2006–2011	Total 2012–2014	2012	2013	2014
All committals to all prisons in Ireland (remand and sentenced episodes, males and females)	87,570	48,916	17,026	15,735	16,155
Male remand committals to all prisons in Ireland	34,323	10,148	3543	3256	3349
Male remand committals to Cloverhill (all screened)	20,084	6177	2248	1953	1976
As percentage of male remand committals to all prisons in Ireland (95 % CI)	58.5 % (58.0–59.0)	60.9 % (59.9–61.8)	63.4 % (61.8–65.0)	60.0 % (58.3–61.7)	59.0 % (57.3–60.7)
Number assessed and taken onto PICLS caseload	3195	1109	374	375	360
As percentage of total male remands to Cloverhill (95 % CI)	15.9 % (15.4–16.4)	18.0 % (17.0–18.9)	16.6 % (15.1–18.2 %)	19.2 % (17.5–21.0)	18.2 % (16.5–20.0)

Committal numbers from Irish Prison Service Annual Reports 2012–2014

Remand committal episodes defined as committals on remand, trial, deportation and extradition

Table 2 shows that 18.0 % (1109/6177) of remand episodes were taken onto the PICLS caseload, with at least one psychiatric assessment by PICLS team members during the years 2012–2014. Proportions of new remands assessed were similar over the 3 years, at 16.6 % in 2012 (374/2248), 19.2 % in 2013 (375/1953) and 18.2 % in 2014 (360/1976).

Over the 3 years 2012-2014, there were 3682 “face to face” assessments of 917 individuals remanded on 1109 occasions. There were 1109 first and 2573 repeat assessments. For those who screened positive on reception or were identified subsequently, committal episodes were followed by a median of two assessments by pairs of team members (range 1–68, mean 3.32, SD 5.2) per committal episode. 505 (45.5 %, 95 % CI 42.6–48.5 %) were seen on one occasion only. There were 10,504 case note entries by PICLS team members, reflecting other activities such as gathering of collateral information, preparation of pre-admission reports, letters to other agencies and other documentation.

### Case-mix: demographic, clinical and offending variables

#### Gender, age, nationality and homelessness

Demographic, clinical and offending variables are summarised in Table 3. All remands were male. For 917 individuals assessed during the period 2012–2014 the mean age at time of first committal was 32.8 years (SD 10.5 range 18–80). For remand episodes (N = 1109) the mean age was 32.6 years (SD 10.2). Among individuals remanded at least once, 84.2 % (772/917) had Irish nationality. Of these, 9.3 % (72/772) identified as Irish travellers, with 9.6 % (88/917) from other EU countries and 6.2 % (57/917) from non-EU countries. One third of all individuals seen (308/917) were homeless at the time of first committal during the three-year study period.

**Table 3:** Case mix: demographic, clinical and offending variables (based on screening, assessment and collateral) for all individuals remanded and taken onto PICLS caseload (N = 917) and remand episodes taken onto PICLS caseload (N = 1109) for three-year period 2012–2014. Similar variables for remand episodes taken onto PICLS caseload for preceding six-year period 2006–2011, with 95 % confidence limits for proportions

Variable	Status at first remand episode for persons taken onto PICLS caseload during 2012–14 (N = 917)			All remand episodes taken onto PICLS caseload during 2012–2014 (N = 1109)			All remand episodes taken onto PICLS caseload during 2006–2011 (N = 3195)		
	No. positive	Percentage	95 % CI limits for percentage	Proportion positive	Percentage	95 % CI limits for percentage	Proportion positive	Percentage	95 % CI limits for percentage
Irish nationality	772	84.2	81.7–86.5	952	85.8	83.7–87.8	2690	84.2	82.9–85.4
Homeless	308	33.6	30.5–36.7	388	35.0	32.2–37.9	748	23.4	22.0–24.9
Lifetime Psychosis	252	27.5	24.6–30.5	339	30.6	27.9–33.4	943	29.5	27.9–31.1
Active psychotic symptoms	192	20.9	18.3–23.7	251	22.6	20.2–25.2	561	17.6	16.3–18.9
History substance misuse	781	85.2	82.7–87.4	954	86.0	83.8–88.0	2773	86.8	85.6–87.9
History deliberate self-harm	571	62.3	59.0–65.4	715	64.5	61.6–67.3	Figure not available		
Violent index offence	329	35.9	32.8–39.1	384	34.6	31.8–37.5	Figure not available		
History of contact with psychiatric service outside prison	599	65.3	62.1–68.4	770	69.4	66.6–72.1	Figure not available		
Age at committal	Mean age 32.8 S.D. 10.5			Mean age 32.6 S.D. 10.2			Mean age 31.8 S.D. 10.8		

Homelessness: defined as not having regular accommodation, rough sleeping or residence in homeless shelters at the time of or during committal

Active psychotic symptoms: defined as hallucinations, delusions and/or thought disorder

Violent offence defined as an act of physical violence on a person and included homicide, assault, robbery, aggravated burglary, contact sexual offences, false imprisonment, driving offences involving injury to others and arson where there was a possibility of injury to others

#### Lifetime psychosis, substance misuse and deliberate self-harm

Based on collateral information, over a quarter of individuals assessed by PICLS (252/917) had a lifetime history of psychotic illness. Of all remand episodes assessed, 69.4 % (95 % CI 66.6–72.1 %) had previous contact with psychiatric services outside prison (770/1109), while for individuals at the time of first committal during the three-year study period, 65.3 % (95 % CI 62.1–68.4 %) had previous contact with psychiatric services outside prison (599/917). Of individuals assessed by the service over the study period, 85.2 % (781/917) had a lifetime history of substance misuse difficulties at the time of first committal during the period 2012–2014. Two thirds (571/917) of had a lifetime history of deliberate self-harm (Table 3).

#### Offence type

Approximately one third (329/917) of individuals remanded and assessed by the service after screening and referral during 2012–2014 were charged with a violent index offence involving physical violence at the time of first committal during this period. A similar proportion of all remand episodes followed by assessment (384/1109) related to charges with a violent offence and 65.4 % (725/1109) with non-violent offences. Most serious index offences for persons remanded and taken onto the PICLS caseload during the years 2012–2014 are summarised in Table 4, below.

**Table 4:** Main index offence type for 1109 remand episodes taken onto PICLS caseload, 2012–2014

Primary index offence	Number	%
Homicide	82	7.4
Assault	128	11.5
Robbery/aggravated burglary	96	8.7
Sexual offences	57	5.1
Arson	12	1.1
False imprisonment	5	0.5
Harassment/stalking/threats	30	2.7
Possession of weapons	44	4.0
Burglary, theft, handle stolen property, tax and fraud offences	271	24.4
Breach of barring, protection or safety order	72	6.5
Public order offences, criminal damage, trespass	190	17.1
Driving offences	35	3.2
Drugs offences	45	4.1
Extradition requests/international arrest warrants	15	1.4
Immigration offences	18	1.6
Failure to appear/contempt of court/other non-violent offences	9	0.8
Total	1109	100.0

PICLS Prison Inreach and Court Liaison Service

#### Current primary diagnosis

Table 5 shows Primary ICD-10 diagnoses at final outcome (discharge, transfer or diversion) for remand episodes assessed by the PICLS team during 2012–2014.

**Table 5:** Primary diagnoses at point of discharge/transfer/diversion for all remand episodes (N = 1109) assessed by the PICLS team from 2012 to 2014

	Primary ICD-10 diagnosis	Number	%
F00–09	Organic disorders	17	1.5
F10–19	Substance abuse disorders	426	38.4
F20–29	Schizophreniform disorders	255	23.0
F30–39	Mood disorders 46/117 (39.3 %) bipolar disorder	117	10.6
F40–59	Neurotic disorders, behavioural syndromes	7	0.6
F60–69	Personality disorders	200	18.0
F70–79	Mental retardation	14	1.3
F80–98	Developmental/childhood disorders	9	0.8
	No mental illness/adjustment reaction	64	5.8
	Total	1109	100.0

*PICLS* Prison Inreach and Court Liaison Service

Over one quarter of all remand episodes taken onto the PICLS caseload had primary ICD diagnoses of schizophrenia, schizotypal and delusional disorders (255/1109) or bipolar affective disorder (46/1109) at the time of final outcome. This represented 4.9 % (95 % CI 4.3–5.4) of all remand episodes (301/6177).

Of the 1109 committal episodes, 38.4 % (426/1109) received a primary diagnosis of substance misuse disorders, 18.0 % (200/1109) personality disorder, 2.7 % (30/1109) other conditions and 5.8 % (64/1009) were assessed as having no mental illness or adjustment issues.

#### Active psychotic symptoms

Among remands from 1st January 2012 to 31st December 2014, 4.1 % overall (251/6177; 95 % CI 3.6–4.6) were identified as having active psychotic symptoms following committal. Of these, 78.5 % received a primary diagnosis of schizophreniform disorders (ICD-10 F20-29: 197/251; 95 % CI 72.9–83.4 %), 12.7 % Bipolar Disorder (ICD-10 F31: 32/251; 95 % CI 8.9–17.5 %) and 8.8 % other, mainly substance-induced or other organic ICD-10 diagnosis (22/251; 5.6–13.0 %).

### Transfer of care

#### Discharges and transfers

Eighty two percent (5068/6177) did not require psychiatric assessment after screening and review of case notes and referrals by members of the PICLS team. Of those identified as requiring psychiatric assessment, 8.8 % (546/6177) were discharged the care of the prison general practitioner or addiction services following one or more assessments. Six (0.1 %) were deported or extradited and six (0.1 %) remained on the PICLS case load as at 9th April 2015, while 3.3 % (202/6177) were referred to the care of in-reach psychiatry teams in other prisons following transfer.

#### Diversion from prison to Mental Healthcare

**Fig. 1:**
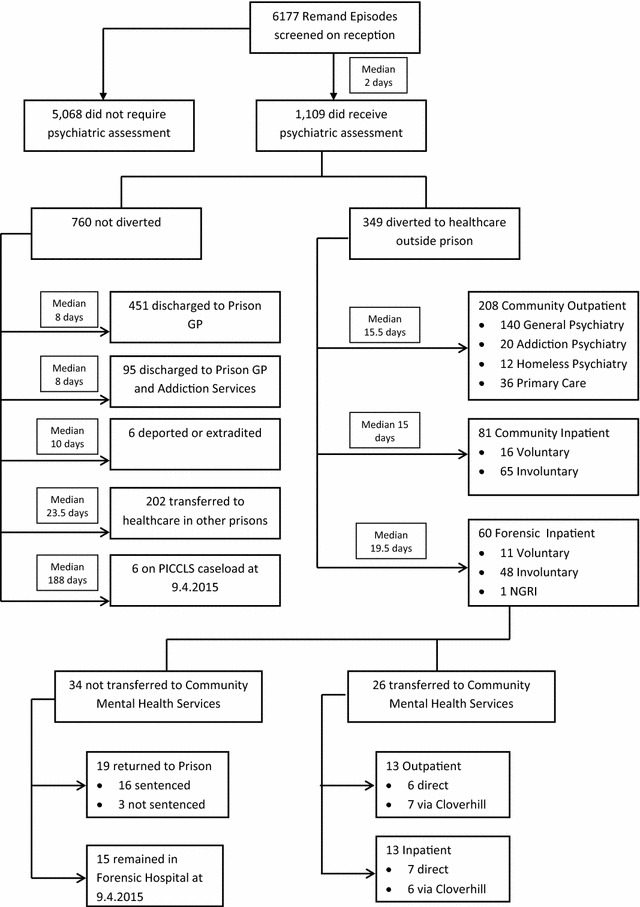
Service mapping: flow diagram from remand to final mental health disposal for 6177 consecutive male remands 2012–2014 with median times from reception in remand prison to mental health transfer

Among remands from 1st January 2012 and 31st December 2014, 5.6 % overall (349/6177, 95 % CI 5.1–6.3) were diverted to healthcare settings outside prison. Of these, 1.0 % (60/6177) were diverted to forensic admission at the Central Mental Hospital, 1.3 % (81/6177) were admitted to general psychiatric hospitals. Psychiatric follow up was arranged in non-inpatient settings for 3.4 % (208/6177) of committal episodes. Of these 208 diversions to community outpatient settings, 140 (67.3 %) were to general psychiatric outpatient departments, 36 (17.3 %) to primary care services and 32 (15.4 %) to specialised addiction psychiatry services, residential rehabilitation centres or psychiatric services for the homeless (Table 6, Fig. 1).

**Table 6:** Time to healthcare outcome: from date of committal, and date of first assessment for all remand episodes (N = 1109) assessed by the Prison Inreach and Court Liaison Service (PICLS), 2012–2014

Outcome	N	Days from committal to outcome				Days from first assessment to outcome			
		Median	Range	Mean	95 % CI	Median	Range	Mean	95 % CI
Discharge to prison GP	451	8.0	0–346	29.3	24.3–34.4	0.0	0–344	12.9	9.4–16.4
Discharge to prison GP and addiction services	95	8.0	1–307	24.0	14.7–33.4	0.0	0–141	9.8	5.2–14.4
Overseas prison transfer	6	10.0	2–24	12.3	2.7–21.9	1.5	0–12	4.0	–1.1–9.1
Community outpatient diversion	208	15.5	0–398	36.7	28.2–45.2	11.0	0–269	26.8	21.4–32.1
General admission	81	15.0	2–60	19.7	16.4–23.0	13.0	0–59	16.8	13.5–20.1
Forensic admission	60	19.5	1–774	52.0	22.4–81.5	17.0	0–773	47.4	17.9–77.0
Transfer to in-reach psychiatry service in other Prison	202	23.5	0–538	54.2	42.7–65.8	17.0	0–538	43.8	33.7–53.8
Remained on PICLS caseload as at 9th April 2015	6	188.0	35–227	160.0	87.6–232.1	187.0	31–225	158.2	84.8–231.6
Total	1109	13.0	0–774	35.9 (SD 65.8)	31.8–40	6.0	0–773	23.7 (SD 53.7)	20.4–27

*GP* general practitioner; *PICLS* Prison Inreach and Court Liaison Service

### Risk-appropriateness of diversions

#### Psychosis and violence

Among the 60 admissions to forensic hospital, 60 % (36/60, 95 % CI 46.5–72.4 %) had been charged with a violent index offence compared with 10 % (8/81, 95 % CI 4.4–19.5 %) of admissions to General Hospitals and 17 % (35/208, 95 % CI 12.0–22.6 %) of diversions to community outpatient treatment settings.

Active psychotic symptoms were identified after committal in 87 % (52/60, 95 % CI 75.4–94.1 %) of forensic admissions, compared with 98 % (79/81, 95 % CI 91.4–99.7 %) of those admitted to general hospitals and 26 % (55/208, 95 % CI 20.6–33.0 %) of community outpatient diversions

Half of the admissions to forensic hospital (30/60, 95 % CI 36.8–63.2 %) were found to have both active psychotic symptoms following committal and had been charged with a violent index offence compared with 10 % (8/81, 95 % CI 4.4–19.5 %) of admissions to General Hospitals and 4 % (8/208, 95 % CI 1.7–7.4 %) of diversions to community outpatient treatment settings.

#### DUNDRUM-1 triage security and DUNDRUM-2 triage urgency scores

Mean DUNDRUM-1 triage security and DUNDRUM-2 triage urgency scores for diversions to forensic, general and community outpatient settings over the years 2012–2014 are summarised in Table 7, together with the total (DUNDRUM-1 plus DUNDRUM-2 scores) for each of these three groups.

**Table 7:** Risk appropriateness of all diversions to healthcare outside prison (N = 349), 2012–2014. Mean DUNDRUM triage security scores (D1) and triage urgency scores (D2), with 95 % confidence intervals for means

		D-1 triage security score		D-2 triage urgency score		Total (D-1 + D-2) triage score	
	N	Mean (SD)	95 % CI	Mean (SD)	95 % CI	Mean (SD)	95 % (CI)
Forensic admission	60	2.39 (0.07)	2.25–2.53	2.01 (0.07)	1.89 –2.14	2.26 (0.06)	2.15–2.37
General admission	81	1.44 (0.05)	1.35–1.53	1.19 (0.06)	1.07–1.31	1.36 (0.05)	1.26–1.45
Outpatient diversions	208	0.77 (0.03)	0.71–0.82	0.26 (0.02)	0.23–0.30	0.59 (0.02)	0.55–0.63

Mean DUNDRUM-1 Security (D-1), DUNDRUM-2 Urgency (D-2) and mean total triage (D-1 + D-2) scores for all admissions to forensic hospitals, general psychiatric admissions and outpatient diversions (N = 349) from 2012 to 2014. D-1 ANOVA F = 327.6, df = 2, p < 0.001; D-2 ANOVA F = 522.6, df = 2, p < 0.001. Combined ANOVA F = 506.9, df = 2, p < 0.001

DUNDRUM-1 triage security mean scores for those diverted to a forensic hospital were in keeping with a medium secure level of need (mean score 2.39, SD 0.07, n = 60), while those admitted to general psychiatric units had a mean score in keeping with acute low security (mean score 1.44, SD 0.05, n = 81) and those diverted to outpatient settings in the community had the lowest mean scores (mean score 0.77, SD 0.03, n = 208) ANOVA F = 327.6, df = 2, p < 0.001. DUNDRUM-2 urgency scores were also consistently higher for those admitted to higher levels of therapeutic security. Confidence intervals did not overlap.

### Efficiency

#### Time to first assessment

The median time from committal to first post-screening assessment by PICLS for the 1009 remand episodes from 1st January 2012 to 31st December 2014 was 2 days. For persons found to have active psychotic symptoms after committal, the median time to assessment was 2 days. For those not identified as having active psychotic symptoms, the median time to assessment was 3 days.

#### Time to diversion

Times from dates of committal and first clinical assessment to healthcare outcome are summarised in Fig. 1 and Table 7. For the 1.0 % (60/6177) receiving forensic admission to the Central Mental Hospital, median time to diversion was 19.5 days from committal and 17 days from first assessment. Median time to diversion was 15.0 days from committal and 13 days from first assessment for those diverted to general psychiatric inpatient units. For those diverted to community outpatient mental health settings, median time to diversion was 15.5 days from committal and 11 days from first assessment.

#### Productivity

Over the study period there were 5.4 whole-time equivalents (WTE) assigned to the service and a further housing support worker who joined the team in January 2014. These posts were not all filled at all times. The 1109 remands taken onto the case load constituted 68 (205/5.4/3) remand episodes case-managed per WTE per year.

### Self-harm

For persons remanded during 2012–2014, there were 70 incidents of self-harm recorded during 48 individual remand episodes, with a range of 1–5 incidents per episode of remand. Thus 0.8 % of remand episodes (48/6177) were followed by one or more acts of self harm in the prison (95 % CI 0.6–1.0), with a rate of 1.1 % acts of self harm recorded per committal episode (70/6177, 95 % CI 0.9–1.4) and 0.9 % of individuals remanded to Cloverhill were recorded as having harmed themselves on one or more occasions during remand episodes (48/5472, 95 % CI 0.7–1.2).

### Service mapping

Final disposal outcomes are described in the transfer of care results section above and presented in flow-chart format in Fig. 1, together with the time to achieve assessment and diversion outcomes. Also presented are subsequent outcomes for forensic admissions.

For those 60 remands transferred to the forensic hospital, 11 were admitted as voluntary patients and 48 as involuntary patients under the Criminal Law (Insanity) Act 2006 [28]. One individual was admitted having been found not guilty by reason of insanity. Of the 60 forensic admissions, 15 remained in the forensic hospital as at 9th April 2015, 3 were discharged to sentenced prisons, 7 were discharged and admitted to general psychiatric hospitals, 6 were discharged to general community psychiatry outpatient services, 29 returned to Cloverhill Prison.

For those 29 returned to Cloverhill Prison, 3 were found not to have a major mental disorder, 13 were transferred to sentenced prisons, 6 were released and admitted to general psychiatric hospitals and 7 were released and follow-up arranged with general community psychiatry outpatient services.

## Testing

### Comparison of clinical activity and outcomes for timeframe 2012–2014 with preceding 6 year timeframe 2006–2011

Table 2 shows that the proportions of remands nationally who were committed to Cloverhill during 2012–2014 was similar to the previous six-year sample 2006–2011 at approximately 60 %. The proportion of remands to Cloverhill taken onto the caseload following screening and referral was higher for the recent period at 18.0 % compared with 15.9 % for the earlier sample.

Table 3 shows that both samples were all male. Mean age at remand was similar for the two periods at 32.6 (2012–14) and 31.8 (2006–2011). For both periods patients assessed were predominantly Irish and had histories of substance misuse. Approximately 30 % of remand episodes taken onto the caseload for both time-periods had a lifetime history of psychosis. A greater proportion of the 2012–2014 caseload was identified as homeless, over one third compared with under one-quarter.

Table 8 shows that for the period 2012–2014 the proportion identified as having active symptoms of psychosis was greater at 4.1 % compared with the earlier period. Proportions and actual numbers per annum diverted to forensic inpatient settings, general inpatient settings and outpatient facilities were all greater for the later period. Confidence intervals did not overlap.

**Table 8:** Service outcomes from 2012 to 2014: proportion assessed, Identification of psychosis and diversion to healthcare. For each column of remands, the number of remands committed and screened (N1) is the denominator for calculation of percentages and confidence intervals

	Total 2006–2011	2012	2013	2014	Total 2012–2014
Number of remands committed and screened (N1)	20,084	2248	1953	1976	6177
Number taken onto PICLS caseload (N2)	3195	374	375	360	1109
Number identified as having active psychotic symptoms	561	79	89	83	251
Number admitted to forensic Hospital	89	18	28	14	60
Number admitted to General Hospital	164	20	32	29	81
Number diverted to community outpatient facilities	319	58	66	84	208
*Number admitted to any hospital (General or forensic)*	*252*	*38*	*60*	*43*	*141*
*Number diverted to any location (forensic hospital, general hospital or community outpatient facilities)*	*572*	*96*	*126*	*127*	*349*
Remands					
Number taken onto PICLS caseload	3195	374	375	360	1109
Percentage (95 % CI)	15.9 % (15.4–16.4)	16.6 % (15.1–18.2)	19.2 % (17.5–21.0)	18.2 % (16.5–20.0)	18.0 % (17.0–18.9)
Number identified as having active psychotic symptoms	561	79	89	83	251
Percentage (95 % CI)	2.8 % (2.6–3.0)	3.5 % (2.8–4.4)	4.6 % (3.7–5.6)	4.2 % (3.4–5.2)	4.1 % (3.6–4.6)
Number admitted to forensic Hospital	89	18	28	14	60
Percentage (95 % CI)	0.44 % (0.36–0.55)	0.74 % (0.44–1.17)	1.43 % (0.96–2.07)	0.71 % (0.39–1.19)	0.97 % (0.74–1.25)
Number admitted to General Hospital	164	20	32	29	81
Percentage (95 % CI)	0.82 % (0.70–0.95)	0.82 % (0.50–1.27)	1.64 % (1.12–2.31)	1.47 (0.99–2.10)	1.31 % (1.04–1.63)
Number diverted to community outpatient facilities	319	58	66	84	208
Percentage (95 % CI)	1.59 (1.42–1.77)	2.39 % (1.82–3.08)	3.38 (2.62–4.28)	4.25 (3.41–5.24)	3.37 (2.93–3.85)
*Number admitted to any hospital (General or forensic)*	*252*	*38*	*60/1953*	*43*	*141*
*Percentage (95* *% CI)*	*1.26* *% (1.11*–*1.42)*	*1.57* *% (1.11*–*2.14)*	*3.07* *% (2.35*–*3.94)*	*2.18 (1.58*–*2.92)*	*2.28* *% (1.93*–*2.69)*
*Number diverted to any location (forensic hospital, general hospital or community outpatient facilities)*	*572*	*96*	*126*	*127*	*349*
*Percentage (95* *% CI)*	*2.85* *% (2.62*–*3.09)*	*3.95 (3.21*–*4.81)*	*6.45* *% (5.40*–*7.63)*	*6.43 (5.39*–*7.60)*	*5.65* *% (5.09*–*6.26)*
*Caseload*					
Number taken onto PICLS caseload (N2)	3195	374	375	360	1109
Number identified as having active psychotic symptoms	561	79	89	83	251
Percentage (95 % CI)	17.56 % (16.25–18.92)	21.12 % (17.10–25.62)	23.73 % (19.52–28.37)	23.06 % (18.80–27.76)	22.63 % (20.20–25.21)
Number admitted to forensic Hospital	89	18	28	14	60
Percentage (95 % CI)	2.79 % (2.24–3.42)	4.81 (2.88–7.50)	7.47 % (5.02–10.61)	3.89 % (2.14–6.44)	5.41 % (4.15–6.91)
Number admitted to General Hospital	164	20	32	29	81
Percentage (95 % CI)	5.13 % (4.39–5.96)	5.35 % (3.30–8.14)	8.53 % (5.91–11.83)	8.06 % (5.46–11.36)	7.30 % (5.84–9.00)
Number diverted to community outpatient facilities	319	58	66	84	208
Percentage (95 % CI)	9.98 % (8.97–11.08)	15.51 % (11.99–19.58)	17.60 % (14.08–21.78)	23.33 % (19.06–28.05)	18.76 % (16.50–21.18)
*Number admitted to any hospital (General or forensic)*	252	38	60	43	141
*Percentage (95* *% CI)*	7.89 % (6.98–8.88)	10.16 % (7.29–13.68)	16.00 % (12.44–20.11)	11.94 % (8.78–15.75)	12.71 % (10.81–14.82)
*Number diverted to any location (forensic hospital, general hospital or community outpatient facilities)*	572	96	126	127	349
*Percentage (95* *% CI)*	17.90 % (16.59–19.28)	25.67 % (21.32–30.41)	33.60 % (28.83–38.63)	35.28 % (30.34–40.46)	31.47 % (28.74–34.30)

For each column of caseload, the number taken onto PICLS caseload (N2) is the denominator

*PICLS* Prison Inreach and Court Liaison Service; *95* *%* CI 95 % confidence interval for proportion

Active psychotic symptoms were identified in a greater proportion of the 2012–2014 caseload at 22.6 % compared with the earlier timeframe at 17.6 %. The proportion of the caseload for whom diversion was achieved to inpatient and outpatient settings was greater for the later period, although 95 % confidence intervals overlapped for diversions to local psychiatric hospitals. Absolute numbers diverted were increased.

Some data were incomplete for the 2006–2011 timeframe, so it was not possible to compare times to assessment and diversion, self-harm and offending information which were not presented in the earlier paper. Similarly, DUNDRUM scores were not available for all of the 2006–2011 cohort, as the instrument was developed during this period.

### Multivariate analysis of 2012–2014 caseload cohort

Binary Logistic regression (method ‘enter’) was used to determine the relative strengths of association with the outcome, first with any psychiatric admission as the outcome, then with any mental health diversion as the outcome. Table 9 also shows the results for ‘any admission’ as outcome. Overall the model correctly predicted 79.2 % of psychiatric diversions. Hosmer and Lemshow tests were acceptable.

**Table 9:** Demographic, clinical and offending variables for male remand committals taken onto PICLS caseload 2012–2014: proportions positive for variables for diversion and non-diversion outcomes

	Diversion outcome					Binary logistic regression ‘enter’ (any psychiatric admission versus no psychiatric admission)			Binary logistic regression ‘enter’ (any diversion versus no diversion)		
	Forensic admission	General admission	Outpatient diversion	Not diverted	Total	Odds ratio	p	95 % CI	Odds ratio	p	95 % CI
N	60	81	208	760	1109						
Psychotic	52 (86.7 %) (75.8–93.1)	79 (97.5 %) (91.4–99.3)	55 (26.4 %) (20.9–32.8)	65 (8.6 %) (6.8–10.8)	251 (22.6 %) (20.3–25.2)	53.42	<0.001	20.47–139.44	6.27	<0.001	3.82–10.29
Known to services	53 (88.3 %) (77.8–94.2)	70 (86.4 %) (77.3–92.2)	179 (86.1 %) (80.7–90.1)	468 (61.6 %) (58.1–65.0)	770 (69.4 %) (66.7–72.1)	1.08	0.84	0.51–2.32	2.45	<0.001	1.66–3.63
Irish	46 (76.7 %) (64.6–85.6)	61 (75.3 %) (64.9–83.4)	189 (90.9 %) (86.2–94.1)	656 (86.3 %) (83.7–88.6)	952 (80.0 %) (77.6–82.2)	0.54	0.08	0.27–1.08	1.01	0.96	0.64–1.61
Homeless	28 (46.7 %) (34.6–59.1)	32 (39.5 %) (29.6–50.4)	87 (41.8 %) (35.3–48.6)	241 (31.7 %) (28.5–35.1)	388 (35.0 %) (32.2–37.8)	0.72	0.21	0.44–1.19	1.01	0.94	0.74–1.39
ICD-10 F20–31	49 (81.7 %) (70.1–89.4)	76 (93.8 %) (86.4–97.3)	72 (34.6 %) (28.2–41.5)	104 (13.7 %) (11.4–16.3)	301 (27.1 %) (24.6–29.8)	2.55	0.03	1.10–5.90	1.83	0.01	1.12–2.91
Substance misuse	48 (80.0 %) (68.2–88.2)	64 (79.0 %) (68.9–86.5)	183 (88.0 %) (82.9–91.7)	659 (86.7 %) (84.1–88.9)	954 (86.0 %) (83.9–87.9)	0.57	0.13	0.27–1.18	0.63	0.05	0.39–1.01
History of Deliberate Self Harm	30 (50.0 %) (37.7–62.3)	33 (40.7 %) (30.7–51.6)	149 (71.6 %) (65.2–77.3)	503 (66.2 %) (62.7–69.5)	715 (64.5 %) (61.6–67.2)	0.75	0.25	0.45–1.23	1.09	0.64	0.77–1.53
Violent offence	36 (60.0 %) (47.4–71.4)	8 (9.9 %) (5.1–18.3)	35 (16.8 %) (12.4–22.5)	305 (40.1 %) (36.7–43.7)	384 (34.6 %) (31.9–37.5)	1.91	0.02	1.09–3.32	0.51	<0.001	0.37–0.72

Odds ratios and 95 % confidence intervals for binary logistic regression ‘enter’

Using forward likelihood ratio as the method and any diversion as outcome yielded a model in four steps consisting of ‘active psychotic symptoms’ (OR 6.4, 95 % CI 3.9–10.3), ‘known to psychiatric services’ (OR 2.4, 95 % CI 1.6–3.5), ‘lifetime diagnosis of bipolar or schizophreniform disorder (ICD10 F20–31)’ (OR 1.8 95 % CI 1.1–2.8) and ‘violent index offence’ (OR 0.52 95 % 0.37–0.73). The model held when backward logistic regression analysis was performed. Of note, when ‘any psychiatric admission’ was used as the outcome for forward likelihood ratio method, some differences emerge. The model correctly predicted 89.4 % of outcomes. The items remaining in the model are ‘psychotic at time of assessment’ (OR 53.5 95 % CI 21.3–134.6), ‘Irish’ (OR 0.41 95 % CI 0.22–0.77), lifetime diagnosis of of bipolar or schizophreniform disorder (ICD10 F20–31) (OR 2.4 95 % CI 1.1–5.5) and ‘violent offence’ (OR 1.9, 95 % CI 1.1–3.3).

Of note, Non-Irish ethnicity was significantly associated with an increased likelihood of psychiatric admission following diversion when controlling for confounding variables. The same results were found when backward logistic regression analysis was performed.

## Discussion

We have shown that data can be routinely collected as part of our service’s normal clinical governance and that this data yields service evaluation. We have adopted a stress-testing model of evaluation and formulated this according to the acronym “STRESS-Testing”. The proportion of remands nationally committed to Cloverhill remained similar over time, at approximately 60 %. The proportion of remands taken onto the PICLS caseload following two-stage screening and referral increased marginally over the two time periods from 16 to 18 % of all remands. Absolute numbers diverted per annum increased between the two time periods. We have shown that year on year the service was able to achieve the intended health gains. At present levels of service resource and population demand, diversions to mental health services were in keeping with epidemiological expectations [9] for rates of active psychosis. The matching of numbers with psychosis detected by systematic screening and numbers diverted may represent a measure of effectiveness while the confidence intervals for these numbers may represent an estimate of capacity. Should demand rise beyond the limits of the confidence intervals described, evidence of service stress might include longer delays before diversion or emerging evidence of cases missed.

The service does not exclude minor mental illnesses or those with substance misuse problems who constituted the great majority of the caseload. The service does regard the ability to identify those with the most severe conditions as its key role. We use the presence or absence of psychotic symptoms as a key measure. This has the advantage of being definable in terms of presence or absence, rather than degree. The proportion of remands to Cloverhill identified as having active psychotic symptoms was higher for the more recent period. Both proportions were within the confidence limits found in the previous study by Curtin et al. which used research diagnostic criteria for diagnosis of an epidemiologically representative sample [9]. This was reassuring in that previous research has found that routine clinical services tended to under-identify severe mental illness among prisoners [26, 27] leading to failure to arrange transfer of care and diversion [2, 28].

The proportion of persons identified as homeless was greater for the later period than for the earlier period. This represents a psychosocial stressor from society at large impacting on patients in the community and in prisons. The addition of a housing support worker represents a service response to this stressor.

Coid and Ulrich [5] predicted that increased prioritisation of psychotic prisoners would have resourcing implications for local and secure psychiatric services, particularly were there to be evidence of increased prevalence rates of psychosis in prisons. Like Fazel and colleagues [3], they identified the need for longitudinal data.

Chow and Priebe [29] described a general reduction in inpatient beds across Western Europe between 1990 and 2012, while forensic beds and prison populations increased. In Ireland they demonstrated that while prison beds increased and general beds reduced between 1990 and 2012, forensic bed numbers per 100,000 inhabitants remained static over the period 2004–2011 at around 2 per 100,000, considerably less than most other developed countries in Western Europe. These represent likely sources of service stress. It may be helpful for similar research to include comparisons of the longitudinal prison in-reach data such as presented in this paper with admission rates to forensic and general psychiatric hospitals and committal rates to prison which in this paper were derived from annual reports of the Irish Prison Service [30–32]. It is beyond the scope of this paper to test such relationships, which may provide information about sources of stress on prison mental health in-reach services.

Coid and Ulrich [5] also described the need to maintain public confidence in diversion arrangements. We have not presented data regarding recidivism in a paper based on routine mental health data collection. DUNDRUM toolkit scores indicated that patients were diverted to appropriate levels of care based on validated measures of need for therapeutic security and urgency of need. DUNDRUM toolkit scores differentiated between forensic, general and outpatient diversions..

Regarding efficiency, a majority of patients taken onto the caseload were formally assessed after screening within 2 days of committal. The rates of case-management per team member and times to assessment and transfer of care provide a basis for comparison with other services.

Hawton and colleagues [33] have described the largest study of self-harming rates in prison settings. They found predicted rates of 5 % self-harming per remand committal episode, albeit with considerable differences between areas and evidence of clustering in time and location. The rates found for this sample were relatively low. Elements of service structure may have been protective. These included transfer of those identified on or following committal as at-risk to the high support unit on D2 wing, mainly in shared accommodation.

We have described arrangements for transfer of care for our sample and mapped these arrangements with the time taken to achieve outcomes. The ability to ‘count in and count out’ is important for any system to operate safely and measurably, including airline checklists and surgical systems [34, 35].

### Limitations

The sample consisted of male remands only. Among female prisoners higher prevalence rates of mental illness have been found in multiple studies [6], and prioritisation requirements may differ. This sample consisted of three-fifths of all male remands nationally, with the remaining two-fifths being remanded to mixed remand-sentenced facilities in the more sparsely populated west of the country. Countries with full national databases may be able to provide more comprehensive data. However the quality of data in this study is consistent and shows good agreement with national epidemiological studies [9] even though routine datasets tend to under-identify persons with psychiatric illnesses.

Clearly, arrangements for transfer of care are not the same as achieving health gains [36]. Previous research [37] described poor rates of engagement with general psychiatric services following release. The admission outcomes presented here describe outcomes achieved. Although the service did not have direct admission rights to general psychiatric beds as recommended by some [38], such admissions were successfully arranged by consensus with local services. We will present outcome data for successful prison transfers and community outpatient diversions in the next paper in this series.

## Conclusions

- Routine data can be collected reliably over time by prison in-reach psychiatry teams over sustained periods, even in locations of greatest ‘stress’ and turnover.
- A model has been described for presenting such data, which may enable detection of systems which are under stress, and suggest areas of recalibration.

## Abbreviations

PICLS: Prison Inreach and Court Liaison Service
CMH: Central Mental Hospital
GP: general practitioner
95 % CI: 95 % confidence interval

## References

[1] Fazel S, Baillargeon J (2011). The health of prisoners. Lancet.

[2] Pierzchniak P, Purchase N, Kennedy H (1997). Liaison between prison, court and psychiatric services. Health Trends.

[3] Fazel S, Hayes AJ, Clerici M, Trestman R (2016). Mental health of prisoners: prevalence, adverse outcomes, and interventions. Lancet Psychiatry Online.

[4] Brugha T, Singleton N, Meltzer H, Bebbington P, Farrell M, Jenkins R, Coid J, Fryers T, Melzer D, Lewis G (2005). Psychosis in the community and in prisons: a report from the British National Survey of psychiatric morbidity. Am J Psychiatry.

[5] Coid J, Ullrich S (2011). Prisoners with psychosis in England and Wales: diversion to psychiatric inpatient services?. Int J Law Psychiatry.

[6] Fazel S, Seewald K (2012). Severe mental illness in 33,588 prisoners worldwide: systematic review and meta-regression analysis. Br J Psychiatry.

[7] Linehan SA, Duffy DM, Wright B, Curtin K, Monks S, Kennedy HG (2014). Psychiatric morbidity in a cross-sectional sample of male remanded prisoners?. Ir J Psychol Med.

[8] Maden T (2000). Psychiatric Morbidity Among Prisoners in England and Wales: By N. Singleton, H. Meltzer & R. Gatward. Br J Psychiatry.

[9] Curtin K, Monks S, Wright B, Duffy D, Linehan S, Kennedy HG (2014). Psychiatric morbidity in male remanded and sentenced committals to Irish prisons. Ir J Psychol Med.

[10] Pakes F, Winstone J (2010). A site visit survey of 101 mental health liaison and diversion schemes in England. J Forensic Psychiatry Psychol.

[11] Pillai K, Rouse P, McKenna B, Skipworth J, Cavney J, Tapsell R (2016). From positive screen to engagement in treatment: a preliminary study of the impact of a new model of care for prisoners with serious mental illness. BMC Psychiatry.

[12] Department of Health. The Bradley report [Internet]. London; 2009. http://www.rcpsych.ac.uk/pdf/Bradleyreport.pdf.

[13] Department of Health, Reed J. Review of mental health and social services for mentally disordered offenders and others requiring similar services. London; 1993. Report no.: Cm. 2088.

[14] Georgiou M, Souza R, Holder S, Stone H, Davies S. Standards for Prison Mental Health Services, quality network for Prison Mental Health Services [Internet]. London: 2015. Royal College Psychiatrists publication number CCQI202. http://www.rcpsych.ac.uk/pdf/Standards%20for%20Prison%20Mental%20Health%20Services%20Publication1.pdf.

[15] Quagliariello M (ed.) (2009). Stress-testing the Banking System. Cambridge: Cambridge University Press. Cambridge books online <http://dx.doi.org/10.1017/CBO9780511635618. Accessed 29 Aug 2016.

[16] Nelson WB (2004). Accelerated testing—statistical models, test plans, and data analysis.

[17] McInerney C, Davoren M, Flynn G, Mullins D, Fitzpatrick M, Caddow M, Caddow F, Quigley S, Black F, Kennedy HG, O’Neill C (2013). Implementing a court diversion and liaison scheme in a remand prison by systematic screening of new receptions: a 6 year participatory action research study of 20,084 consecutive male remands. Int J Mental Health Syst.

[18] Armonk NY. IBM SPSS Statistics for Windows, 20th edn. IBM Corporation; 2011.

[19] DasGupta A, Cai TT, Brown LD (2001). Interval estimation for a binomial proportion. Stat Sci.

[20] Grubin D, Carson D, Parsons S. Report on new prison reception health screening arrangements: the results of a pilot study in 10 prisons. Newcastle. http://a1538.g.akamai.net/7/1538/13355/v001/homeoffice.download.akamai.com/13355/Doc/1011/101112067.pdf. Accessed Oct 2002.

[21] Kennedy HG, O’Neill C, Flynn G, Gill P. Dangerousness, understanding, recovery and urgency manual (the Dundrum quartet), 1st edn. http://www.tara.tcd.ie/bitstream/handle/2262/39131/THE%20DUNDRUM%20TOOLKIT%20V1%200%2021%201304101.pdf?sequence=1&isAllowed=y. Accessed Mar 2010.

[22] International statistical classification of diseases and related health problems, 10th revision (ICD-10). Geneva; 1992.

[23] Flynn G, O’Neill C, McInerney C, Kennedy HG (2011). The DUNDRUM-1 structured professional judgment for triage to appropriate levels of therapeutic security: retrospective-cohort validation study. BMC Psychiatry.

[24] Freestone M, Bull D, Brown R, Boast N, Blazey F, Gilluley P (2015). Triage, decision-making and follow-up of patients referred to a UK forensic service: validation of the DUNDRUM toolkit. BMC Psychiatry.

[25] Flynn G, O’Neill C, Kennedy HG (2011). DUNDRUM-2: prospective validation of a structured professional judgment instrument assessing priority for admission from the waiting list for a forensic mental health hospital. BMC Res Notes.

[26] Birmingham L, Mason D, Grubin D (2000). Identifying mental illness at reception into prison. Crim Behav Mental Health.

[27] Reed J, Lyne M (1997). The quality of health care in prison: results of a programme of semi-structured inspections. Br Med J.

[28] Birmingham L (2001). Diversion from custody. Adv Psychiatr Treat.

[29] Chow WS, Priebe S (2016). How has the extent of institutional mental healthcare changed in Western Europe? Analysis of data since 1990. BMJ Open..

[30] Irish prison service. Irish prison service annual report 2012 [Internet]. 2012. http://www.irishprisons.ie/images/pdf/annualreport2012web.pdf.

[31] Irish prison service. Irish prison service annual report 2013 [Internet]. 2013. http://www.irishprisons.ie/images/pdf/ar_2013.pdf.

[32] Irish prison service. Irish prison service annual report 2014 [Internet]. 2014. http://www.irishprisons.ie/images/pdf/ar2014_english.pdf.

[33] Hawton K, Linsell L, Adeniji T, Sariaslan A, Fazel S (2014). Self-harm in prisons in England and Wales: an epidemiological study of prevalence, risk factors, clustering, and subsequent suicide. Lancet.

[34] WHO. Surgical safety checklist (First edn). World Health Organisation. http://www.who.int/patientsafety/safesurgery/tools_resources/SSSL_Checklist_finalJun08.pdf?ua=1.

[35] WHO. WHO guidelines for safe surgery 2009. Safe surgery saves lives. http://whqlibdoc.who.int/publications/2009/9789241598552_eng.pdf. Accessed 8 Sep 2016. 23762968

[36] Hopkins G (2014). Thornicroft G Engagement with mental health services on release from prison. J Forensic Res.

[37] Lennox C, Senior J, King C, Hassan L, Clayton R, Thornicroft G, Shaw J (2012). The management of released prisoners with severe and enduring mental illness. J Foren Psychiatry Psychol.

[38] James D (1999). Court diversion at 10 years: can it work, does it work and has it a future?. J Foren Psychiatry Psychol.

